# Diagnóstico de hemoglobinopatías en el laboratorio clínico: hallazgo de una hemoglobina hofu oculta en HPLC

**DOI:** 10.1515/almed-2024-0081

**Published:** 2025-03-31

**Authors:** Maitane Echeverría Urroz, Ana Isabel López Delgado, Raquel Oliveros Conejero, David Álvarez Nistal

**Affiliations:** Laboratorio Bioquímica, 16650 Hospital Universitario Donostia, Donostia, España

**Keywords:** hemoglobina Hofu, hemoglobinopatía, serie roja

## Abstract

**Objetivos:**

Las hemoglobinopatías son trastornos que afectan a la estructura, función y/o producción de hemoglobina. Obedecen a mutaciones o deleciones en los genes codificantes para la síntesis de globina. La presentación clínica es muy variable, desde formas asintomáticas hasta anemia grave. Para su diagnóstico es necesario el estudio mediante pruebas de laboratorio.

**Caso clínico:**

En este artículo, se presenta el caso de una paciente que consulta por astenia. Debido a antecedentes familiares de hemoglobinopatía, se realiza estudio de eritropatías. La cromatografía líquida de alta resolución (HPLC) muestra un perfil normal de distribución de hemoglobinas. El estudio mediante electroforesis capilar de zona en medio alcalino, en cambio, pone en evidencia un pico de migración rápida sin identificar. Tras estudio genético, se detecta una mutación en el gen HBB causante de hemoglobinopatía Hofu.

**Conclusiones:**

a hemoglobina variante Hofu es una hemoglobina levemente inestable. En formas heterocigotas es habitualmente asintomática, pero en combinación con otras alteraciones puede causar anemia. Característicamente, el tiempo de retención de la Hb Hofu en HPLC es muy cercano al de la Hb A(0) y a menudo eluyen juntas. Por tanto, su detección puede quedar enmascarada y conllevar a errores en la interpretación del estudio.

## Introducción

Las hemoglobinopatías son un conjunto de alteraciones de la hemoglobina que se presentan con clínica diversa dónde el estudio mediante pruebas de laboratorio es imprescindible en la orientación diagnóstica. El laboratorio clínico dispone de diferentes métodos para la separación, cuantificación y detección de hemoglobinas variante y talasemias. En este artículo presentamos mediante un caso clínico el hallazgo de una hemoglobina variante rara, la hemoglobina Hofu.

## Caso clínico

Mujer de 45 años de edad, originaria del norte de la Península Ibérica, que consulta por clínica de astenia continuada en atención primaria. Como antecedentes de interés, destacan los antecedentes familiares por diagnóstico de talasemia no filiada tanto por parte paterna como materna.

Desde atención primaria se solicita analítica con hemograma básico, bioquímica y determinación de HbA_2_ y HbF debido a antecedentes familiares de talasemia. En los resultados se objetiva anemia microcítica de tipo ferropénica (Tabla 1). Ante la sospecha de una posible eritropatía hereditaria asociada a anemia ferropénica, se informa de la importancia de corregir la ferropenia antes de realizar el estudio de posibles eritropatías, ya que la ferropenia puede disminuir la cantidad de HbA_2_. Una vez instaurado el tratamiento y corregida la ferropenia, se solicita un nuevo estudio completo con solicitud de muestra de suero (BD Vacutainer SST II Advance) y plasma (BD Vacutainer K_2_E (EDTA)). En el hemograma se observa un aumento en el valor de hemoglobina alcanzando el límite inferior del intervalo de referencia, junto con microcitosis y una concentración de HCM por debajo del valor de referencia (Tabla 1). Los parámetros de hemólisis (LDH, haptoglobina, bilirrubina total y reticulocitos) se encuentran dentro del intervalo de referencia.

**Tabla 1:** Resultados del perfil de anemia antes y después del tratamiento con hierro oral para corregir la ferropenia. Segunda analítica realizada a los 11 meses.

Prueba, unidades	Situación basal	Paciente tras recibir tratamiento con hierro	Intervalo de referencia
Hemoglobina, g/dL	9,1	12	12-15,3
Eritrocitos, /µL	4,40 × 10^6^	4,88 × 10^6^	3,8-5 × 10^6^
VCM, fL	73	79,9	80–97
ADE, %	17,2	19,5	11,5–15,6
HCM, pg	20,7	24,6	27–33
CHCM, g/dL	28,3	30,8	32–36
Hierro, µg/dL	21	32	30–160
Ferritina, ng/mL	7	20	15–150

**Figura 1:**
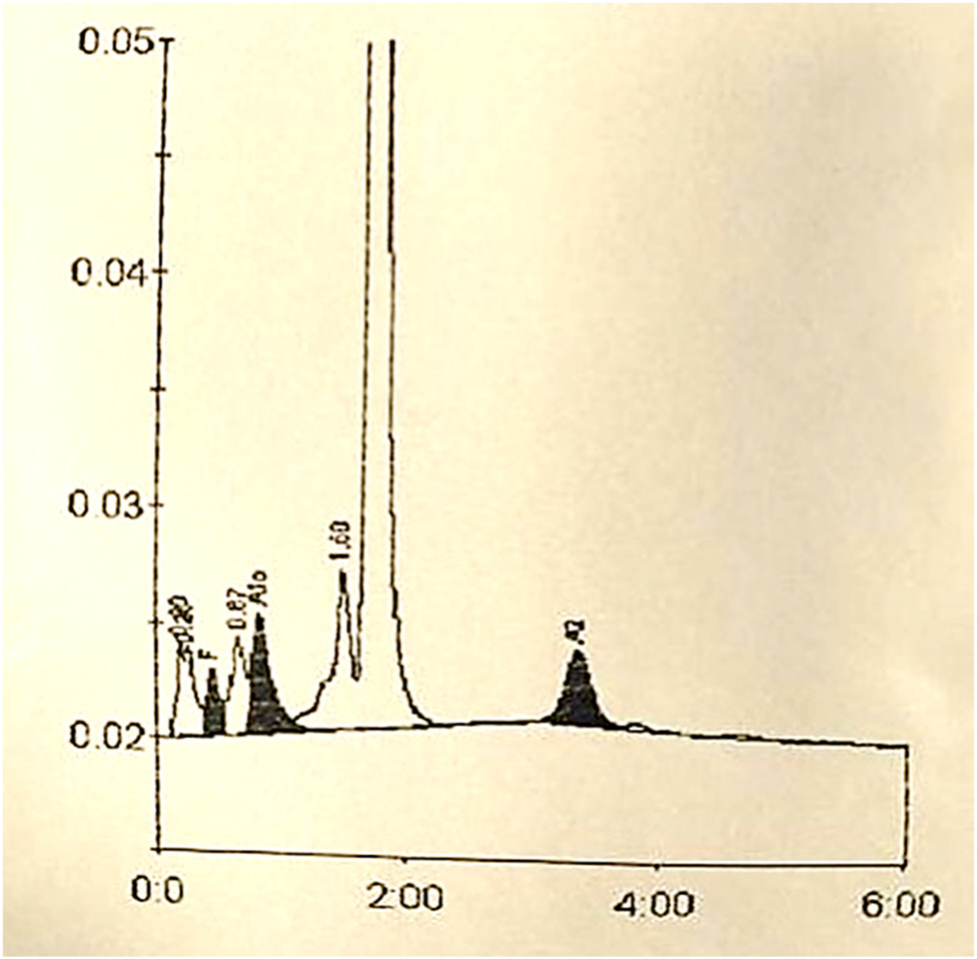
Cromatograma obtenido mediante cromatografía HPLC (BIO-RAD, D-10) acoplado a un detector fotométrico. Resultados expresados en porcentaje y tiempo de retención: HbA: 82,3 %, 1,73 min; HbA_2_: 3,4 %, 3,36 min; HbF: 1,4 %, 0,46 min; HbAc: 5,1 %, 0,84 min.

**Figura 2:**
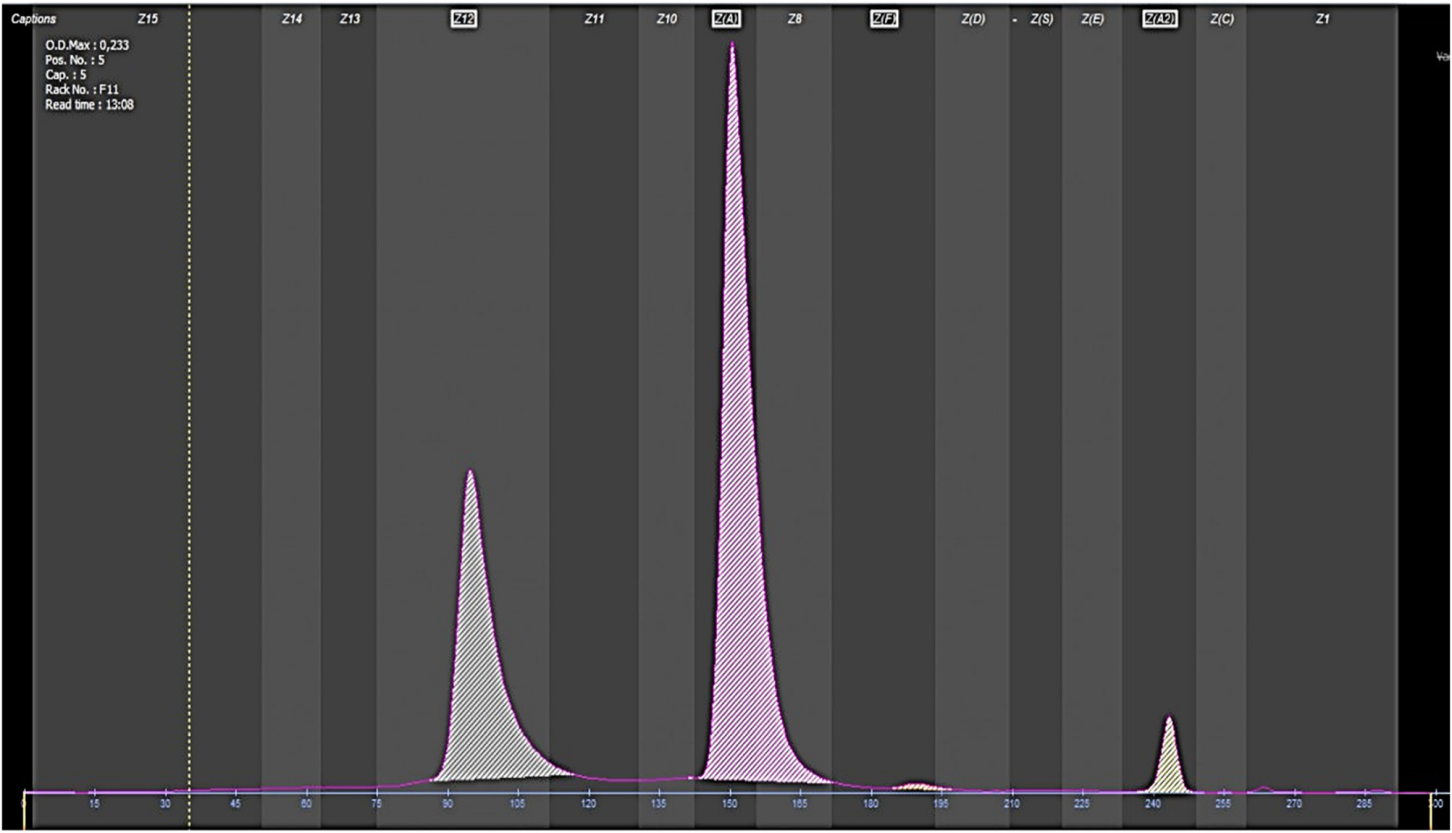
Gráfica resultado de electroforesis capilar (Capillarys 2, Sebia). Observamos picos*.* De izquierda a derecha, el pico gris corresponde al pico sin identificar, 31,9 %. A su derecha, el pico rosa representa a la HbA, 64,2 %. Junto al pico de HbA, un pequeño pico naranja, correspondiente a la HbF. Por último, en el extremo derecho, el pico amarillo donde migra la HbA_2_, 3,4 %.

El estudio de eritropatías se realiza mediante dos métodos diferentes, para así poder detectar posibles hemoglobinas ocultas en uno u otro método. El cromatograma obtenido mediante HPLC (BIO-RAD, D-10), muestra una gráfica (Figura 1) sin presencia de variantes anómalas de hemoglobina. El estudio mediante electroforesis capilar (Sebia, Capillarys 2), en cambio, evidencia un pico correspondiente a una hemoglobina de migración rápida (Figura 2).

Ante la creciente sospecha de hemoglobinopatía, se recomienda valorar el estudio genético y la paciente es derivada a consultas de hematología. Se realiza una revisión de hemogramas previos observándose episodios de anemia ferropénica, trombocitosis reactiva sin aumento en otras series ni citopenias asociadas. Por último, se amplía el estudio con secuenciación mediante método Sanger de los genes de globina alfa y beta.

El estudio molecular evidencia una mutación en el gen HBB, detectándose la variante HBB:c.380T>A (p.Val127Glu) en heterocigosis. Esta mutación ha sido descrita como la causante de Hemoglobina Hofu [1].

Finalmente, la paciente es diagnosticada de hemoglobinopatía Hofu en heterocigosis e inicia control mediante hemogramas periódicos en consultas de hematología, sin presentar clínica.

Actualmente, la familia está pendiente de estudio.

## Discusión

La hemoglobina está formada por cuatro cadenas de globina, dos tipos alfa y dos tipos beta, junto con cuatro grupos hemo. Las variantes de las cadenas alfa y beta se combinan dando lugar a diferentes tipos de hemoglobina durante el crecimiento, con diferente afinidad al oxígeno y efecto cooperativo. Este es un proceso regulado genéticamente [2].

En cuanto a las hemoglobinopatías, son el conjunto de alteraciones hematológicas hereditarias que afectan a las cadenas de globina, siendo las enfermedades monogénicas más prevalentes. Pueden tratarse de una afectación cuantitativa, estructural o funcional de una o varias cadenas de globina.

Se estima que cerca del 7 % de la población mundial es portadora de un gen mutado. Asimismo, cada año nacen entre 300.000 y 500.000 niños con hemoglobinopatías graves homocigotas, siendo las más frecuentes la talasemia y la drepanocitosis [3].

En la actualidad, se ha visto una generalización mundial de las hemoglobinopatías debido al aumento de los movimientos migratorios, convirtiéndose en un problema de salud global emergente con un número cada vez mayor de casos en regiones originariamente no endémicas.

La alteración genética causante de la hemoglobinopatía puede darse en los genes codificantes de las cadenas de globina o en sus regiones regulatorias dando lugar a mutaciones sin sentido, alteración del número de copias, fusiones genéticas, etc. Por lo tanto, es grupo muy heterogéneo de patologías. Pueden clasificarse en dos grandes grupos: las hemoglobinas variantes y las talasemias. En el presente texto nos centraremos en aquellas de tipo variante, en las cuales hay un defecto estructural y en ocasiones funcional.

Las hemoglobinopatías de tipo variante son causadas principalmente por un cambio de aminoácido en la cadena peptídica [4]. El cambio aminoacídico puede derivar en cambios en las propiedades fisicoquímicas de la hemoglobina causando a su vez alteraciones en la estructura, solubilidad, afinidad al oxígeno y en la función fisiológica.

La hemoglobinopatía por hemoglobina variante más frecuente es la causada por la HbS (anemia falciforme), pero se han descrito más de 1.200 variantes de hemoglobina.

Las técnicas disponibles para su detección incluyen: cromatografía líquida de alta resolución (HPLC) de intercambio iónico, electroforesis alcalina y ácida, isoelectroenfoque, electroforesis capilar y métodos de biología molecular. Las guías internacionales recomiendan que, ante la sospecha de una variante significativa de hemoglobina, su presencia debe confirmarse mediante un método alternativo adecuado (grado de evidencia 1A) [5].

En nuestra área de trabajo, el laboratorio de Análisis Clínicos del Hospital Universitario de Donostia, el estudio de hemoglobinopatías se realiza de forma simultánea y no excluyente mediante dos técnicas diferentes: HPLC de intercambio iónico y electroforesis capilar de zona en medio alcalino. Así se dispone de la oportunidad de detectar posibles hemoglobinas anómalas ocultas por otros picos en la cromatografía y viceversa.

La HPLC mediante columna de intercambio catiónico se realiza en el equipo D-10 (Bio-Rad). Las diferentes hemoglobinas son separadas según su interacción iónica con la fase estacionaria mediante un gradiente de tampón con fuerza iónica creciente. La fase móvil junto con los analitos atravesará la célula de flujo del detector fotométrico, obteniéndose una señal de absorbancia medida a una longitud de onda de 415 nm. Esta señal será proporcional a la concentración del analito y eluirá a un tiempo específico y característico de la hemoglobina a estudio.

Por su parte, la electroforesis capilar se realiza en el equipo Capyllarys 2 (Sebia). En este caso, las diferentes fracciones de hemoglobina migrarán según su movilidad electroforética mediante flujo electroendosmótico en tampón alcalino. En pH alcalino, la hemoglobina tiene carga negativa y migra hacia el ánodo (+) [6]. Las diferentes variantes alcanzarán el detector en momentos separados en el tiempo según su movilidad electroforética.

La hemoglobina Hofu fue descrita por primera vez por Miyaji y col. en Japón, cuando realizaban estudios de variantes de hemoglobina en la población del área de la ciudad Hofu. Durante este estudio hallaron una variante de hemoglobina con diferente migración que la HbA a pH alcalino, pero indiferenciable de ésta a pH neutro. En concreto, la nueva hemoglobina se situaba más hacia el ánodo en electroforesis en gel de agarosa a pH 8,6 [7], tratándose de una hemoglobina de migración rápida. A su vez, el estudio genético demostró una sustitución de la valina de la cadena β en la posición 126 por ácido glutámico [7].

Característicamente, puede no separarse correctamente de la HbA dependiendo del método utilizado, como mediante la cromatografía HPLC de intercambio iónico, dónde la separación es parcial. En cambio, en la electroforesis en pH alcalino, al tener una migración más rápida hacia el ánodo, se obtiene una separación correcta de las distintas fracciones [8].

Clínicamente, la presentación heterocigota de la Hb Hofu suele presentarse de forma asintomática o con ligera anemia ya que se trata de una hemoglobina levemente inestable [8]. Es una hemoglobinopatía rara que ha sido hallada en pacientes procedentes de Japón, España, Sri Lanka, India y América. También ha sido descrita en combinación con HbS y beta talasemia [8], pudiendo causar hemólisis en combinación con ésta última. Junto a HbS, habitualmente de presentación asintomática, cuando ha presentado clínica ha sido en forma de crisis dolorosas [9].

En nuestro caso, la paciente presentaba astenia y una ligera anemia que se corregía parcialmente tras tratamiento. El estudio de eritropatías demostró la presencia de una hemoglobina anómala de un 31 % mediante electroforesis capilar de zona en pH alcalino, que no pudo ser separada mediante cromatografía de intercambio catiónico. Según la bibliografía reportada, los porcentajes encontrados de esta hemoglobina en ausencia de talasemia varían entre un 28–31 % [8]. El estudio genético descartó la presencia de una talasemia asociada y de alteraciones en la cadena alfa. La hemoglobina variante fue identificada como Hb Hofu. La paciente continuará en seguimiento por el Servicio de Hematología.

## Puntos de aprendizaje

– Las hemoglobinas variantes muestran una clínica variada, siendo habitualmente asintomáticas en su forma heterocigota. Su diagnóstico es importante ya que en formas homocigotas o en combinación con otras hemoglobinopatías pueden dar lugar a formas más graves.
– Es importante realizar el estudio de posibles hemoglobinas variantes mediante dos métodos diferentes, debido al posible enmascaramiento entre picos.
– La hemoglobina Hofu es una hemoglobinopatía estructural rara causada por una mutación en el gen HBB, siendo esta asintomática en los portadores heterocigóticos de la variante.
